# P19 Total visual loss in connective tissue disease – an unusual presentation of optic peri-neuritis

**DOI:** 10.1093/rap/rkab068.018

**Published:** 2021-10-19

**Authors:** Lucy Parker, Sandeep Mukherjee

**Affiliations:** University Hospitals Dorset, Christchurch, United Kingdom

## Abstract

**Case report - Introduction:**

Since the discovery of aquaporin-4 antibody in 2004, the association between visual loss and auto-immune disease has become increasingly apparent. Certainly, neuro-myelitis optica spectrum disorders (NMOSD) can co-exist with connective tissue disease (CTD). The complexity comes in trying to differentiate the two diagnoses. NMOSD feels like the tip of an iceberg with regards to autoimmune eye disease. When no specific antibody can be found, the clinical syndrome can only be described.

**Case report - Case description:**

A 22-year-old female, with a history of childhood ITP presented with 4 months of myalgia, arthralgia, raised inflammatory markers (ESR 81, CRP 49) and weight loss. She had no clinical features suggestive of CTD. PET-CT demonstrated abnormal uptake associated with large joints. Small joint musculoskeletal ultrasound was normal. Initial ANA was borderline positive. 3 months later she presented with right-sided retro-orbital pain of 3 weeks' duration, followed by abrupt onset total visual loss in the right eye. The visual acuity in the left eye was also reduced. Ophthalmological examination revealed brilliant white optic atrophy of the right disc, with only perception of light. On the left, the disc and acuity were normal.

MRI coronal STIR images of the orbits demonstrated inflammatory change in the peri-optic structures and signal change in the optic nerve anteriorly. Post-contrast images confirmed abnormal enhancement affecting the right optic nerve sheath and surrounding fat, some intrinsic enhancement of the optic nerve and some ill-defined enhancement of the left optic nerve sheath. Saggital T2 imaging of the spine confirmed no cord lesions.

All disease-specific antibodies were negative (rheumatoid factor, CCP, connective tissue disease screen, dsDNA, ENA panel, anti-phospholipid, centromere, MOG, Aquaporin-4). HLA-B27 was negative. Complements were normal. HIV, syphilis and Lyme serology were all negative. She was initially treated with oral prednisolone and hydroxychloroquine with poor recovery of vision in the right eye.

2 years later, she developed a small deep left thalamic infarct, and was switched to azathioprine.

Following a further episode of right eye pain associated with a rise in ESR, treatment was changed to injectable methotrexate resulting in resolution of arthralgia and normalisation of inflammatory markers.

Surveillance MRI this year has identified a subtle area of high FLAIR signal in the right pons, with further interval scan awaited.

**Case report - Discussion:**

Inflammatory eye disease is a developing field, with the relatively recent identification of aquaporin-4 and MOG antibodies in the context of neuromyelitis optica spectrum disorders.

**Case report - Key learning points:**

This patient initially presented with an inflammatory arthritis, and then went on to develop abrupt onset visual loss. The rheumatologist should remain vigilant for development of optic peri-neuritis in CTD, and consider investigation if eye pain or discomfort develops.

In NMOSD, aquaporin-4 (AQP4) antibodies are directed against the AQP4 channels on astrocytes. It is possible that optic peri-neuritis in this case may be due to inflammation in the optic nerve sheath caused by an as-yet unidentified antigen/antibody-mediated process.

In patients presenting with CTD who develop optic neuritis or peri-neuritis, further evaluation for NMOSD is essential. AQP4 and MOG antibodies are now readily available and easily checked in routine practice.

There appears to be an emerging association between NMOSD and CTD, and the rheumatologist should remain vigilant for the presence of dual pathology.

